# Posterior Chamber Phakic Intraocular Lens Implantation in Eyes with an Anterior Chamber Depth of Less Than 3 mm: A Multicenter Study

**DOI:** 10.1038/s41598-018-31782-y

**Published:** 2018-09-06

**Authors:** Kazutaka Kamiya, Kimiya Shimizu, Akihito Igarashi, Yoshihiro Kitazawa, Takashi Kojima, Tomoaki Nakamura, Kazuo Ichikawa

**Affiliations:** 1 Department of Ophthalmology, University of Kitasato School of Medicine, Kanagawa Sagamihara, Japan; 2 Department of Ophthalmology, Sanno Hospital, Tokyo, Japan; 3 Kobe Kanagawa Eye Clinic, Tokyo, Japan; 4 Department of Ophthalmology, Japanese Red Cross Gifu Hospital, Gifu, Japan; 5 Nagoya Eye Clinic, Aichi Nagoya, Japan; 6 Chukyo Hospital, Aichi Nagoya, Japan

## Abstract

This study was aimed to assess the clinical outcomes of Visian ICL (hole ICL; STAAR Surgical, Inc.) implantation for the correction of myopic refractive errors in eyes having an anterior chamber depth (ACD) below the current manufacturer’s recommendation (<3.0 mm). We comprised 365 eyes of 201 consecutive patients (mean age ± standard deviation, 35.7 ± 7.5 years) with spherical equivalents of −8.66 ± 3.54 D. We evaluated the safety, efficacy, predictability, stability, intraocular pressure (IOP), endothelial cell density (ECD), and complications. The safety and efficacy indices were 1.12 ± 0.22 and 0.98 ± 0.22. At 1 year, 90% and 98% of eyes were within ± 0.5 and 1.0 D of the attempted correction, respectively. Changes in the manifest refraction from 1 week to 1 year postoperatively were −0.08 ± 0.34 D. The mean ECD loss was 0.2 ± 8.7%. No eyes showed a significant ECD loss (≥30%). We found no significant correlation between the ACD and the change in ECD (Pearson correlation coefficient r = −0.048, p = 0.360). No significant IOP rise or vision-threatening complication occurred at any time. These findings indicate that the surgical indication of ICL implantation should be reconsidered in terms of ACD.

## Introduction

The EVO Visian ICL (Hole ICL; KS-AP^TM^; STAAR Surgical Inc, Monrovia, California, USA), a posterior chamber phakic intraocular lens with a central hole, has been widely accepted as one of the viable surgical options for the correction of moderate to high ametropia^1–4^. The manufacturer currently recommends this surgery in eyes with an anterior chamber depth (ACD) of 3.0 mm or more for hole ICL implantation, especially in consideration of corneal endothelial cell density (ECD) safety issues. However, this ACD recommendation, possibly derived from undisclosed data of manufacturer, was not definite, and still controversial, especially among ICL experienced surgeons. Indeed, we encountered some candidates who seek refractive surgery and have an ACD below the manufacturer’s recommendation. This tendency is more prominent in older patients. So far there have been no published studies on the outcomes of ICL implantation in eyes with a relatively low ACD, or on the relationship between ACD and corneal endothelial cell loss. This study may give us basic insights in the expansion of the current surgical indication of ICL implantation in terms of ACD. The purpose of the current study was to retrospectively evaluate the visual and refractive outcomes of hole ICL implantation in eyes having a relatively low ACD in a large cohort of patients presenting at major medical centers, with special attention to corneal ECD.

## Results

### Study Population

Preoperative demographics of the study population are summarized in Table 1. Toric and non-toric ICL models were implanted in 191 eyes (52%) and 174 eyes (48%), respectively. No significant intraoperative complications occurred in any case.

**Table 1:** Preoperative demographics of the study population in eyes with a low anterior chamber depth.

Characteristic	Mean ± Standard Deviation
Age (years)	35.7 ± 7.5 years (95% CI, 21.1 to 50.3 years)
Gender (Male: Female)	M: F = 71: 130
Manifest spherical equivalent (D)	−8.66 ± 3.54 D (95% CI, −1.72 to −15.61 D)
Manifest cylinder (D)	1.29 ± 1.16 D (95% CI, 0.00 to 3.57 D)
Log MAR UDVA	1.36 ± 0.30 (95% CI, 0.78 to 1.94)
Log MAR CDVA	−0.17 ± 0.09 (95% CI, 0.01 to −0.35)
White-to-white distance (mm)	11.4 ± 0.4 mm (95% CI, 10.8 to 12.1 mm)
Anterior chamber depth (mm)	2.85 ± 0.12 mm (95% CI, 2.62 to 2.98 mm)
Central cornea thickness (μm)	534.3 ± 34.1 μm (95% CI, 467.5 to 601.2 μm)
Intraocular pressure (mmHg)	14.2 ± 2.6 mmHg (95% CI, 9.0 to 19.4 mmHg)
Endothelial cell density (cells/mm^2^)	2933.6 ± 342.2 cells/mm^2^ (95% CI, 2262.9 to 3604.3 cells/mm^2^)

CI = confident interval, Log MAR = logarithm of the minimal angle of resolution, UDVA = uncorrected distance visual acuity, CDVA = corrected distance visual acuity.

### Safety and Efficacy

**Figure 1:**
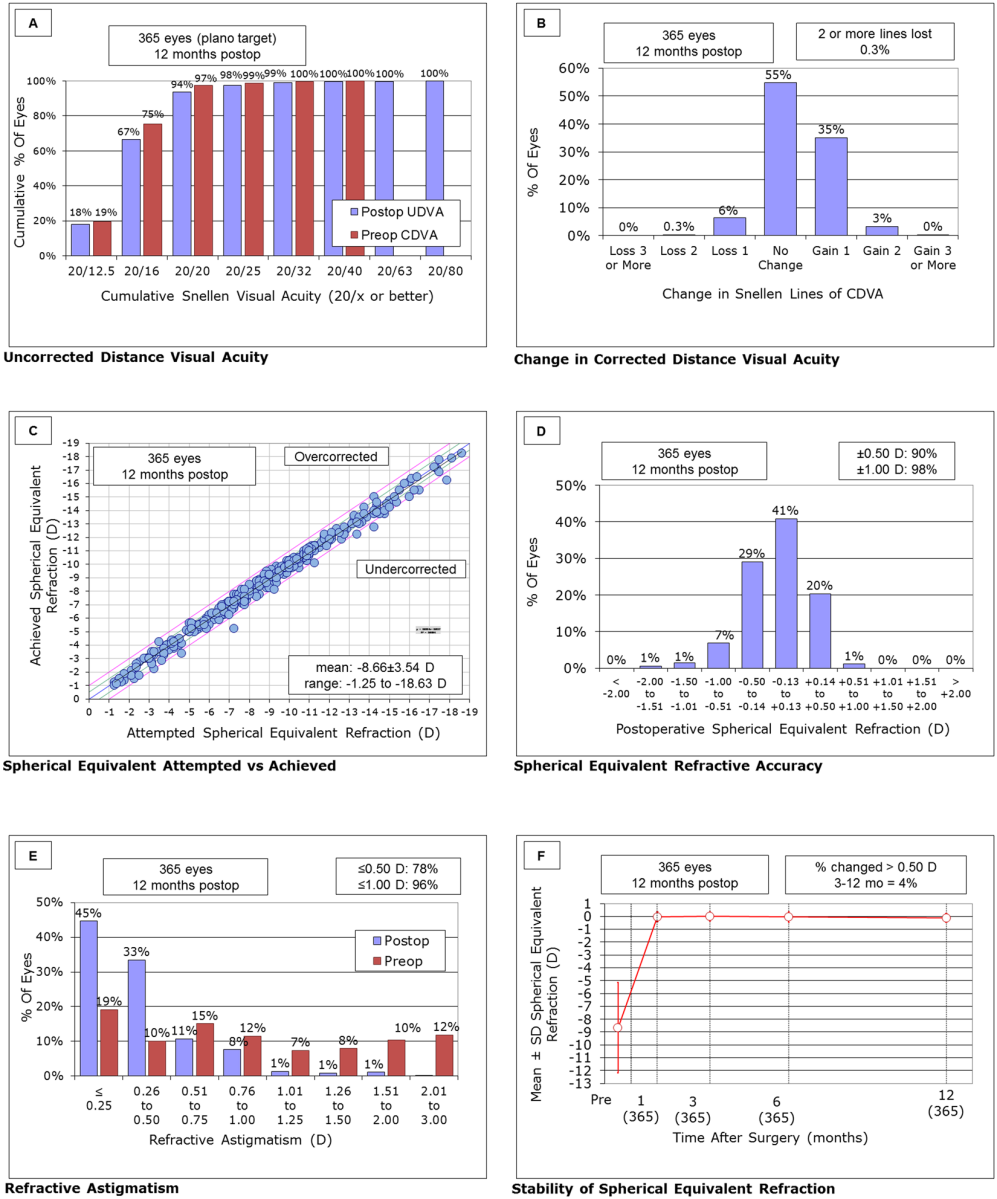
(**A**) Cumulative percentages of eyes attaining specified levels of postoperative uncorrected distance visual acuity (UDVA), (**B**) changes in corrected distance visual acuity (CDVA), (**C**) a scatter plot of attempted vs. achieved correction, (**D**) spherical equivalent refractive accuracy, (**E**) preoperative and postoperative refractive astigmatism, and (**F**) time course of manifest spherical equivalent after hole implantable collamer lens implantation in eyes with an anterior chamber depth (ACD) of <3.0 mm.

The safety and efficacy indices were 1.12 ± 0.22 and 0.98 ± 0.22. Logarithm of the minimal angle of resolution (logMAR) CDVA significantly improved from −0.17 ± 0.09 preoperatively to −0.21 ± 0.08 postoperatively (Student t test, p < 0.001). At 1 year postoperatively, logMAR UDVA also significantly improved from 1.36 ± 0.30 preoperatively to −0.15 ± 0.11 postoperatively (p < 0.001). At 1 year postoperatively, 94%, 98%, and 99% of eyes had UDVA of 20/20 or better, 20/25 or better, and 20/32 or better, respectively (Fig. 1A). Two hundred one eyes (55%) showed no change in CDVA, 127 eyes (35%) gained 1 line, 12 eyes (3%) gained 2 lines, and 23 eyes (6%) lost 1 line, and 1 eye (0.3%) lost 2 lines (Fig. 1B).

### Predictability and Stability

A scatter plot of the attempted versus the achieved manifest spherical equivalent correction, the spherical equivalent refractive accuracy, and the preoperative and postoperative refractive astigmatism and the time-course changes in the manifest spherical equivalent are also shown in Figs. 1C–E. At 1 year postoperatively, 90% and 98% were within ± 0.5 and 1.0 D of the attempted correction, respectively. Changes in the manifest spherical equivalent from 1 week to 1 year postoperatively were −0.08 ± 0.34 D (Fig. 1F).

### Intraocular Pressure

**Figure 2:**
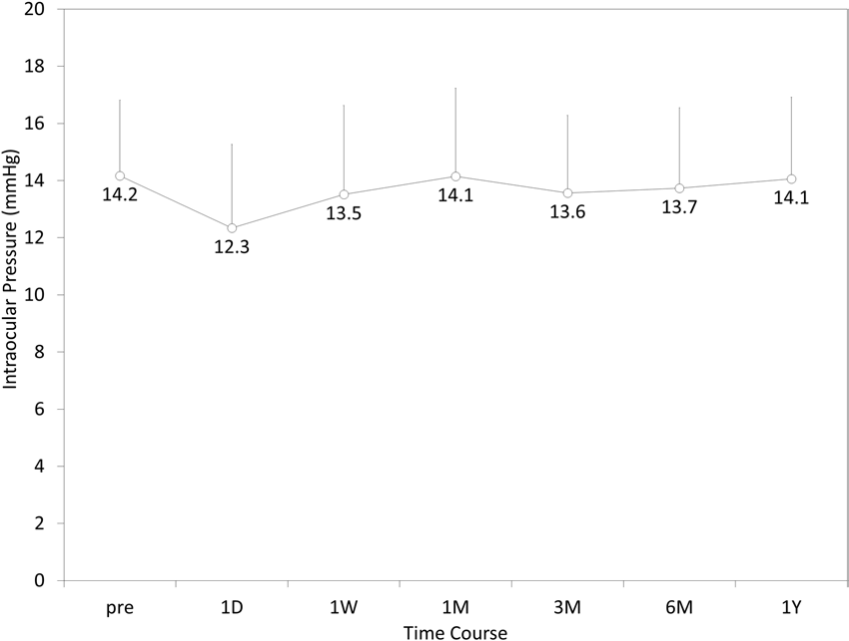
Time course of intraocular pressure after hole implantable collamer lens implantation in eyes with an anterior chamber depth (ACD) of <3.0 mm.

The time-course changes in the IOP are shown in Fig. 2. No significant IOP rise of > 21 mmHg, pigment dispersion syndrome, pupillary block, or angle-closure glaucoma due to the excessive ICL vault, was found at any time in this series.

### Endothelial Cell Density

**Figure 3:**
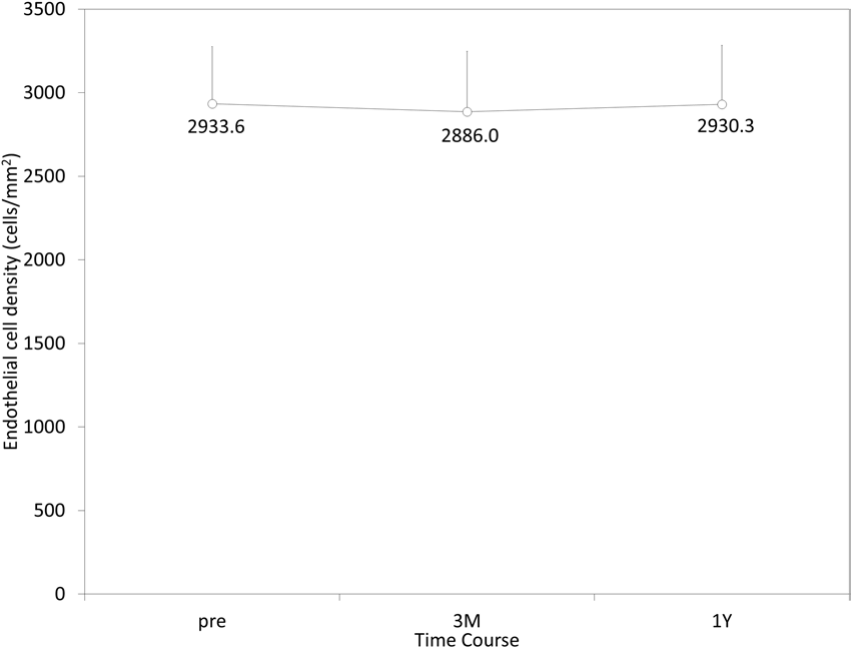
Time course of endothelial cell density after hole implantable collamer lens implantation in eyes with an anterior chamber depth (ACD) of <3.0 mm.

**Figure 4:**
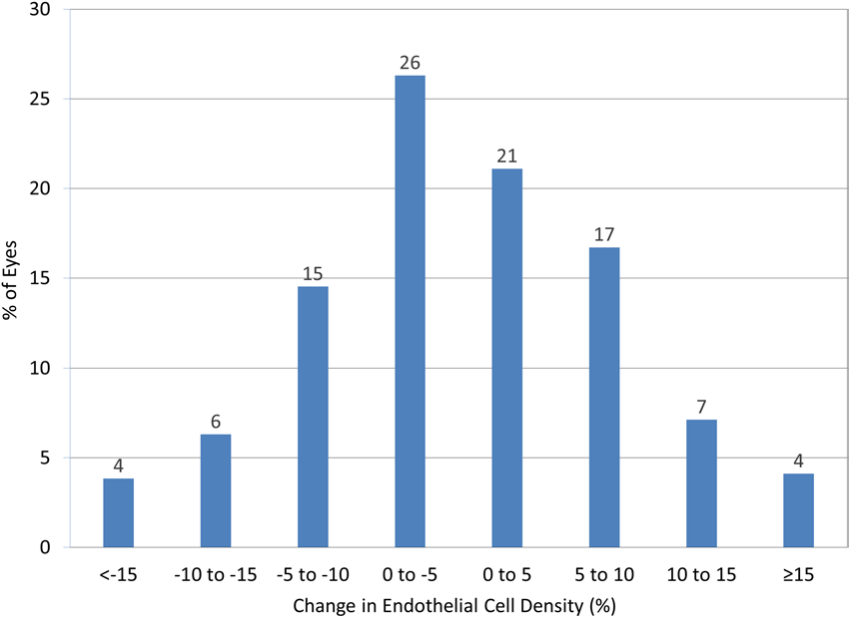
Distribution of corneal endothelial cell loss after hole implantable collamer lens implantation in eyes with an anterior chamber depth (ACD) of <3.0 mm.

**Figure 5:**
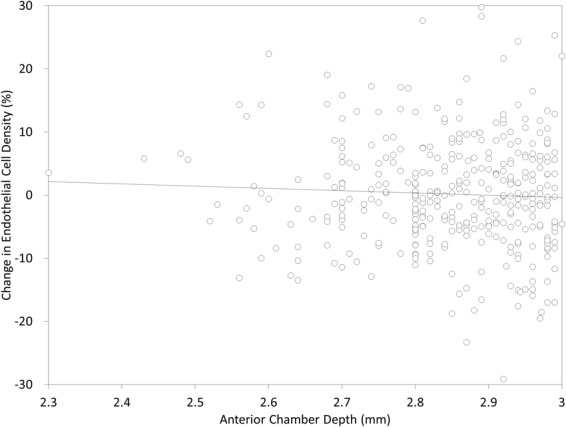
A graph showing no significant correlation between the anterior chamber depth (ACD) and the change in the endothelia cell density (ECD) at 1 year postoperatively (Pearson correlation coefficient r = −0.048, p = 0.360).

The time-course changes in the ECD are shown in Fig. 3. The distribution of the ECD loss at 1 year postoperatively was listed in Fig. 4. The mean ECD loss was 0.2 ± 8.7%. No eyes showed a significant ECD loss (≥30%). We found no significant correlation between the ACD and the change in the ECD at 1 year postoperatively (Pearson correlation coefficient r = −0.048, p = 0.360)(Fig. 5).

### Secondary Surgeries / Adverse Events

Of the 365 eyes, 2 eyes (0.6%) developed significant axis rotation of a toric ICL model (≥30 degrees), and required ICL repositioning to the intended axis. Only 1 eye (0.3%) required ICL extraction and implantation of a larger ICL in order to prevent frequent axis rotation. Otherwise, neither asymptomatic or symptomatic cataract formation, nor other vision-threatening complications occurred at any time throughout the 1-year observation period.

## Discussion

Our multicenter study supports the view that ICL implantation performed well in terms of safety, efficacy, predictability, and stability, and that neither vision-threatening complications such as cataract formation, pupillary block, pigment dispersion syndrome, nor angle-closure glaucoma, occurred in any case, even in eyes where the ACD was below the manufacturer’s recommendation. Although no eyes showed a significant ECD loss of ≥30% or a significant IOP rise of >21 mmHg, we should be aware that ECD loss of 15% or more did occur in approximately 4% of these eyes. As far as we can ascertain, this is the first study to assess the visual and refractive outcomes of ICL implantation in a large cohort of patients having an ACD of <3.0 mm. We believe that this information is clinically meaningful for understanding the actual status of ICL implantation in eyes having a relatively low ACD, because the number of older candidates who seek refractive surgery is expected to increase, now that a new ICL model with a central hole aiming to minimize cataract formation even for such older patients has been introduced.

Our current visual and refractive outcomes of hole ICL implantation in a relatively low ACD (<3.0 mm) were comparable with previous outcomes with a deep ACD^5–14^. The mean ECD loss has been reported to be −1.1 to 8.5% after hole ICL implantation, as listed in Table 2. The ECD loss in the present study was also comparable with, or better than, that in previous studies^5–12,15^. However, ECD loss of 15% or more was found in 4%. These findings indicate that the mean ECD loss was clinically negligible, and that the possible risk of significant ECD loss was considerably low, but may increase, despite good visual and refractive outcomes in such eyes. Although a non-contact specular microscope has been reported to be considerably reproducible for ECD measurements, it cannot be used interchangeably, especially when using automated image analysis software^16–18^. It should also be emphasized that all surgeries were performed by experienced ICL surgeons. Therefore, we do not recommend ICL implantation in eyes with an ACD of <3.0 mm for beginner surgeons who did not yet get accustomed to this surgery.

**Table 2:** Previous studies reporting central endothelial cell density (ECD) loss after hole implantable collamer lens (ICL) implantation.

Author	Year	Eyes	Follow-up	Mean ECD loss (%)
Shimizu *et al*.^5^	2012	20	6 months	2.8
Alfonso *et al*.^6^	2013	138	6 months	8.5
Huseynova *et al*.^7^	2014	44	3 months	−1.1
Lisa *et al*.^8^	2015	147	1 year	1.7
Fernández-Vigo *et al*.^9^	2016	50	3 months	6.8
Shimizu *et al*.^10^	2016	32	5 years	0.5
Liu *et al*.^11^	2016	82	3 months	4.8
Kamiya *et al*.^12^	2017	351	1 year	0.1
Goukon *et al*.^15^	2017	34	2 years	0.3
Current	2018	365	1 year	0.2

ECD = endothelial cell density.

The surgical criteria for the hole ICL surgery at these institutions usually includes unsatisfactory correction with spectacles or contact lenses, age of 21 ≥ years, stable refraction, myopia and myopic astigmatism, ACD of ≥3.0 mm for hole ICL, ECD ≥1800 cells/mm^2^, and no history of ocular surgery, progressive corneal degeneration, cataract, glaucoma or uveitis. With regard to ACD, the manufacturer currently recommends that the minimum ACD is 3.0 mm for hole ICL implantation, since the current ICL model is preserved in a balanced salt solution, whereas it was previously recommended that the minimum ACD was 2.8 mm for conventional ICL implantation, since the previous ICL model was preserved in 0.9% NaCl. This ACD recommendation, possibly derived from undisclosed data by the manufacturer, especially in consideration of ECD safety issues, was not definite, and is still controversial, among ICL surgeons. Indeed, the anterior chamber is deepened, to some extent, by insertion of viscoelastic surgical devices into the anterior chamber, when we perform ICL implantation. We found no significant association between the ACD and a change in the ECD in the present study. Based on our current findings, it is suggested that the surgical indication of ICL implantation should be reconsidered, and may be expanded, especially in terms of ACD.

This study has at least two limitations. One is that our case series were conducted in a retrospective fashion. Since our study may include not only information bias but also selection bias, the evidence level is not very high. Second, we included only those patients who completed a 1-year follow-up in this series. Therefore, there might exist some selection bias in this study. Assuming that patients who were satisfied with visual function tended to be lost to follow-up, the overall visual outcomes might be better than those in the current study.

In conclusion, our multicenter case series showed that ICL implantation was good in all outcomes of safety, efficacy, predictability, and stability, for the correction of myopia and myopic astigmatism, and that no vision-threatening complications such a significant endothelial cell loss occurred in any case, even in eyes with an ACD of <3.0 mm. It is suggested that ICL implantation is a feasible surgical option for the correction of refractive errors even in eyes with an ACD outside of the manufacturer’s recommendation, and that the surgical indication in terms of ACD should be reconsidered, and may be expanded, after accumulation of long-term clinical data for such eyes.

## Methods

### Study Population

The protocol was registered with the University Hospital Medical Information Network Clinical Trial Registry (000030285). Three hundred sixty five eyes of 201 consecutive patients (71 men and 130 women, mean age ± standard deviation (SD), 35.7 ± 7.5 years) who underwent hole ICL implantation at 5 major clinical centers (Kitasato University Hospital, Sanno Hospital, Kobe Kanagawa Eye Clinic, Nagoya Eye Clinic, and Sato Yuya Eye Clinic) from January 2014 to December 2016, and who had an ACD (measured from the corneal endothelium to the anterior surface of the crystalline lens) of <3.0 mm, were retrospectively investigated. Those patients who regularly returned for postoperative examination, and who completed a 1-year follow up, were enrolled in this review of the clinical charts. This study was conducted as a collaborative work of the Japan ICL Study Group. In principle, we selected the toric ICL model when manifest cylinder was 1.5 D or more, and the non-toric ICL model when it was less than 1.5 D. Preoperatively, we measured the white-to-white distance and the ACD with a scanning-slit topography. Preoperatively, and at 1, 3, 6, and 12 months postoperatively, we evaluated uncorrected distance visual acuity (UDVA), corrected distance visual acuity (CDVA), manifest spherical equivalent, and intraocular pressure (IOP) using a non-contact tonometer. We also evaluated the ECD using a non-contact specular microscope preoperatively, and 3 and 12 months postoperatively. We performed slit-lamp biomicroscopic and funduscopic examinations on all eyes. Written informed consent for this surgery was obtained from all patients after explanation of the nature and possible consequences of the study. This retrospective review of the clinical charts was approved by the Institutional Review Board at Kitasato University and followed the tenets of the Declaration of Helsinki. Our Institutional Review Board waived the requirement for informed consent for this retrospective study.

### Lens Power and Size Calculation

The manufacturer provides an online calculator system based on a modified vertex formula, and this was used to determine ICL power. In order to minimize refractive errors, we selected emmetropia as the target refraction in all eyes. By using a nomogram provided by the manufacturer, we selected the proper ICL size, based on ACD and white-to-white distance.

### Surgical Procedure

We previously described the detailed surgical procedures^5,19^. In short, topically anesthetic and dilating agents were administered, and a 3-mm temporal corneal incision was made. After injection of a viscosurgical substance in the anterior chamber, the ICL was gently inserted using an injector. Next, a manipulator was used to fixate the ICL haptics into the ciliary sulcus, all remaining viscosurgical substance was replaced with a balanced salt solution, and a miotic agent was administered. Toric model ICL implantation required preoperative horizontal axis markings. After insertion in the posterior chamber, the toric ICL needed to be rotated with a manipulator to the intended axis. Postoperatively, antibiotic and steroidal medications were topically administered for 2 weeks, 4 times a day.

### Statistical Analysis

All statistical analyses were performed using a statistical software (Bellcurve for Excel, Social Survey Research Information Co, Ltd., Tokyo, Japan). The normality of all data samples was first checked using the Kolmogorov-Smirnov test. Since the use of parametric statistics was possible, the Student t test was used for statistical analysis to compare the pre- and post-treated data, and the Pearson correlation coefficient was used to assess the correlation between the two variables. The results are expressed as mean ± standard deviation, and a p**-**value of less than 0.05 was determined statistically significant.
